# Double-branched stent graft and four-stage deployment in total arch repair: safety and feasibility evaluation in porcine models

**DOI:** 10.1093/icvts/ivae049

**Published:** 2024-03-16

**Authors:** Chenhao Wang, Wenfan Li, Peng Yang, Chen Lu, Yu Zhang, Haiyue Wang, Zhenghua Xiao, Jia Hu

**Affiliations:** Department of Cardiovascular Surgery, West China Hospital, Sichuan University, Chengdu, Sichuan Province, P.R. China; Department of Cardiovascular Surgery, West China Hospital, Sichuan University, Chengdu, Sichuan Province, P.R. China; Department of Cardiovascular Surgery, West China Hospital, Sichuan University, Chengdu, Sichuan Province, P.R. China; Department of Cardiovascular Surgery, West China Hospital, Sichuan University, Chengdu, Sichuan Province, P.R. China; Department of Cardiovascular Surgery, West China Hospital, Sichuan University, Chengdu, Sichuan Province, P.R. China; Department of Cardiovascular Surgery, West China Hospital, Sichuan University, Chengdu, Sichuan Province, P.R. China; Department of Cardiovascular Surgery, West China Hospital, Sichuan University, Chengdu, Sichuan Province, P.R. China; Department of Cardiovascular Surgery, West China Hospital, Sichuan University, Chengdu, Sichuan Province, P.R. China; Department of Cardiothoracic Surgery, West China Guang’an Hospital, Sichuan University, Guang’an, Sichuan Province, P.R. China

**Keywords:** Aortic arch repair, Double-branched stent graft, Preclinical study, Porcine, Type A acute aortic dissection

## Abstract

**OBJECTIVES:**

The primary objective of this research was to evaluate the safety and feasibility of an innovative double-branched stent graft system employing four-stage deployment technology for aortic arch repair in porcine models.

**METHODS:**

The double-branched stent graft system consisted of a proximal polyester artificial blood vessel, the main and double-branched stent grafts and a delivery system. We utilized 12 healthy pigs as experimental animals (6 per group). Postimplantation, samples were collected at 90 and 180 days after the operations. Preoperative and postoperative imaging and intraoperative arterial blood gas analyses were performed. After the pigs were euthanized, the implanted product, surrounding tissue and major organs were collected for pathological analysis.

**RESULTS:**

The technical success rate of the stent graft implants was 100% (12/12). All animals survived to the experimental end point. Perioperative assessments showed intact stent grafts, and imaging features at the end of the follow-up period revealed neither endoleak nor device migration. No major adverse cardiovascular events were observed during the postoperative follow-up period. Pathological examinations confirmed the satisfactory biocompatibility of the stent graft.

**CONCLUSIONS:**

This innovative double-branched stent graft system with four-stage deployment technology was affirmed as a safe and feasible option for aortic arch repair in accordance with our preclinical evaluation with porcine models.

**Figure:**
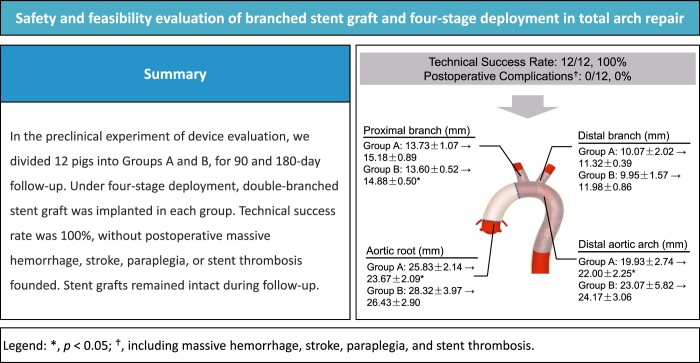

## INTRODUCTION

Aortic diseases, particularly type A acute aortic dissection, are recognized as life-threatening cardiovascular emergencies. Hemi-arch replacement is a reliable option, although it is associated with a reduced occurrence of false lumen thrombosis and a higher incidence of reintervention [1]. Total arch replacement enhances survival and minimizes redo operations, yet it demands significant surgical expertise for delicate grafting and potential risks due to sternotomy, cardiopulmonary bypass (CPB), deep hypothermic circulatory arrest and prolonged selective cerebral perfusion [2–5].

Advanced arch repair endeavours to combine the benefits of conventional surgery and endovascular treatment [6–8]. The Thoraflex Hybrid graft and the E-vita Open Plus are the 2 most widely utilized frozen elephant trunk components of hybrid arch repairs [9, 10]. The mainstream frozen elephant trunks, however, are associated with their own set of challenges, such as type I and type II endoleaks and distal dissections [11, 12]. Notably, triple-branched stent grafts have proved to be a viable therapeutic choice for complete arch repair in recent years [13, 14]. However, the anatomically intricate aortic arch mandates the implanting of various-sized three-branch stents, posing a limitation to the clinical utility of surgical procedures.

We recently developed a double-branched stent graft system and four-stage deployment technology, indicated for comprehensive arch repair under open replacement. Total arch repair can be gracefully accomplished by deploying the stent graft into the proximal descending aorta, the left subclavian artery (LSA), the aortic arch and the left common carotid artery (LCCA), also enabling straightforward execution of proximal and distal vascular anastomoses. Flexible distance between 2 distal neighbouring branches of our device allows adaptation to a wider range of patients, and the four-stage deployment technology ensures improved wall apposition and deployment control. Collectively, these features could enhance the outcomes by reducing complications after the stent graft is implanted. Our goal was to report the early results from our preclinical evaluation involving porcine models implanted with this advanced stent graft and four-stage deployment technology.

## MATERIALS AND METHODS

### Study design

The present study was a preclinical in vivo animal research project that involved implanting aortic stent grafts in animals to assess the device's safety and feasibility. All experimental procedures strictly followed the ARRIVE (Animal Research: Reporting of In Vivo Experiments) guidelines. Furthermore, the study complied with ethical principles for animal experimentation and was approved by the animal experimental ethics review committee (Ethics Approval Number: SS-2020-PHZJ).

### The novel double-branched stent graft and the delivery system

**Figure 1:**
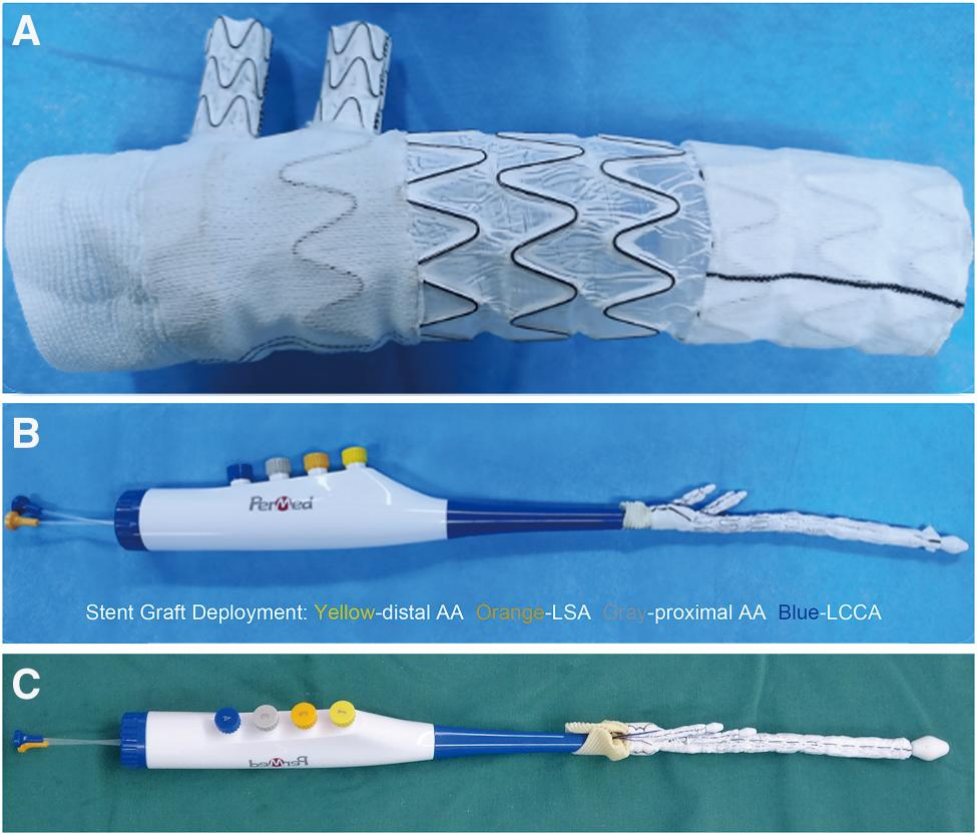
The novel double-branched stent graft system. (**A**) The stent graft is composed of a proximal polyester vascular graft fabric, a distal nickel-titanium alloy bare wire stent, and a polytetrafluoroethylene membrane. The stent comprised a main graft and 2 branched grafts. (**B**, **C**) The delivery system to match the newly designed double-branched stent graft. AA: aortic arch; LCCA: left common carotid artery; LSA: left subclavian artery.

Conceived and fabricated by Permed Biomedical Engineering Co., LTD (Beijing, China), this double-branched stent graft system comprised a proximal polyester artificial blood vessel, the main and double-branched stent graft and a corresponding delivery system (Fig. 1). The supporting body of the covered stent was manufactured using nickel-titanium alloy wire with shape memory properties. The “W” shape structure of the wire rendered the stent both compressible and self-expanding. The main stent graft consisted of the proximal section for effective arch repair and the distal section, acting as the stented elephant trunk. The diameters of the proximal and distal stents, branch length and diameter and distal stent length were designed to be variable. Furthermore, the main graft between the LSA and the LCCA was suitably soft and thin, without alloy wires for support, which allows for transverse folding to facilitate its placement and adjustment during surgical procedures. Both the inner and outer layers of the bare stent were coated with a polytetrafluoroethylene membrane, which exhibited effective blood-blocking properties and had excellent biocompatibility. Proximal and distal ends of the stent were covered with a polyester cloth to promote sealing after implantation and to prevent endoleak. Additionally, a proximal artificial vascular fabric, sutured adjacent to the covered stent, was designed to aid in reducing the risk of postoperative haemorrhage. The delivery system comprised handle parts and release knobs. After pre-installing the stent in the stent-loading area on the delivery device, the stent was sent to the target location by pulling and releasing the cable. The stent automatically sprang open, following which the delivery device was removed. During the operation, the stent was delivered to the target site via the fixed rod after opening the aorta. The stent created an artificial false lumen at the site of injury, isolating the high-pressure blood flow from the lesion site and preventing blood pressure-induced damage.

### Animal preparation

To ensure anatomical and pathological similarity with humans and haemodynamic similarity, we utilized Chinese hybrid Landrace porcine models. As per the draft Guiding Principles for Technical Review of Animal Experimental Studies of Medical Devices from the Medical Device Technical Review Center of the National Medical Products Administration of the People's Republic of China, 12 pigs were used for this study, randomly divided into group A and group B. The animals were individually marked with ear tags for identification. The experimental facilities and the animals were provided by an animal experiment institute (Yinsnake Medical Technology Co., LTD, Guangzhou, China) accredited by the Standardization Administration of China for Laboratory Animals—Requirements of Environment and Housing Facilities. Prior to the operation, the animals were acclimatized in the feeding room. After the device was implanted, the animals were transferred to the care unit for monitoring and were later transferred back to the feeding room once recovered. Each animal was kept in an individual cage and had access to food and water before and after the operation. Water was freely accessible; the feeding, which was based on individual weight, was quantitative, administered twice daily at fixed times. The animal facility room temperature and relative humidity were suitable.

The animals underwent fasting and restricted water intake prior to the initiation of anaesthesia. Prior to skin preparation, Sutaride (VIRBAC, Paris, France) and atropine sulfate were administered intramuscularly to provide sedation. After successful induction of anaesthesia with propofol (Guangdong Jiabo Pharmaceutical, Qingyuan, China), endotracheal intubation was performed, and anaesthesia was maintained using propofol and isoflurane. Mechanical ventilation was used to assist breathing, and intravenous access was established. Additionally, heart rate and blood pressure monitoring, as well as blood gas analysis, were performed.

### Operative procedures and deployment technology

**Figure 2:**
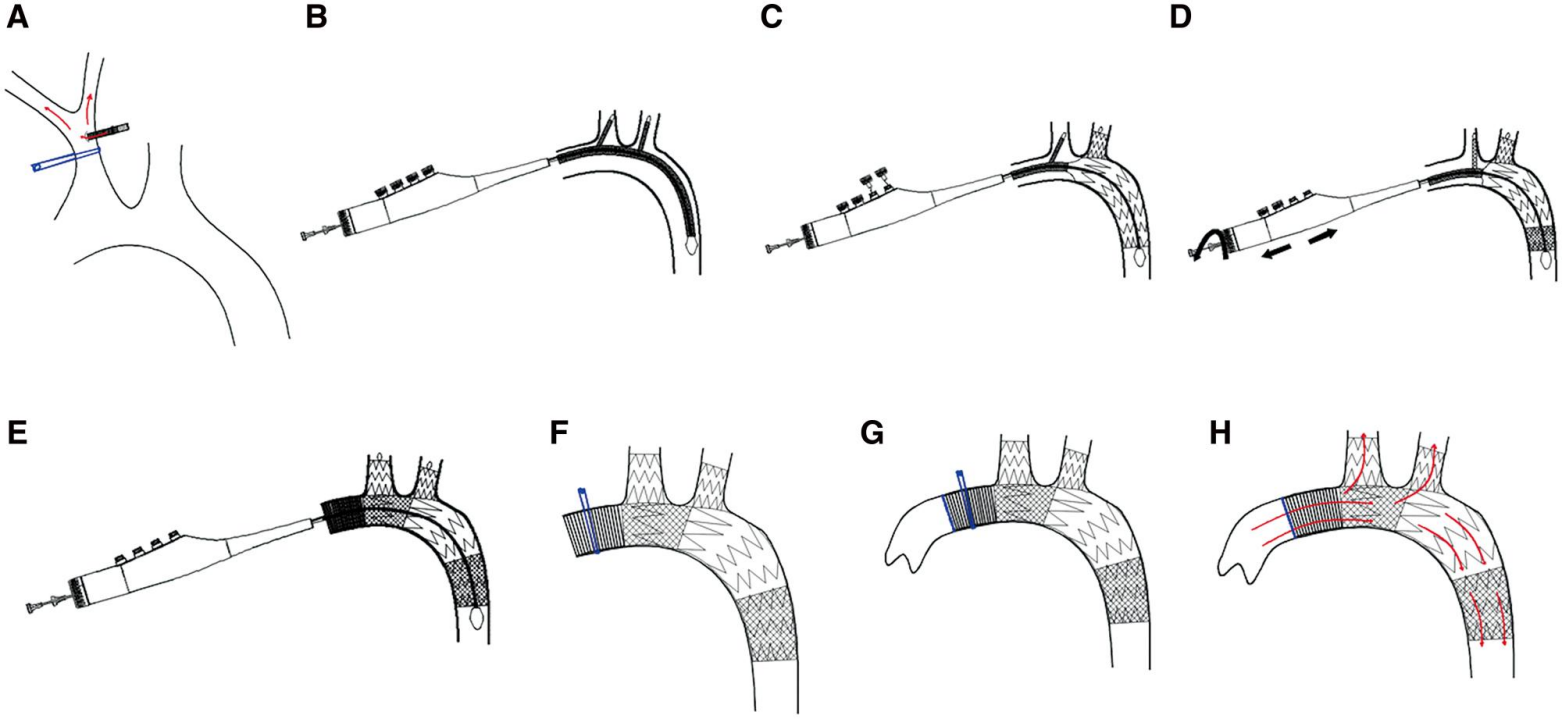
Diagrams of implant procedures for the double-branched stent graft and operation. (**A**) The right innominate artery was clamped and anastomosed laterally as an artificial vessel. (**B**) The double-branched stent graft was placed into the aortic arch. (**C**) The distal main graft and then the distal branch graft were released as the first 2 stages of deployment. (**D**) The position of the second branch stent graft in the right innominate artery was adjusted moderately. (**E**) The proximal main graft and the proximal branch graft were released as the second deployment. (**F**) The delivery system was withdrawn from the aorta. (**G**) The artificial vessel located proximal to the main stent was anastomosed with the ascending aorta. (**H**) The artery blood flow was restored after anastomosing.

The selection of device size was guided by preoperative computed tomography angiography (CTA). Following a routine sternotomy, the proximal portion of the right innominate artery (IA) was meticulously isolated from the surrounding tissue. The activated clotting time of the whole blood was measured to ensure adequate heparinization. The right IA was clamped with lateral forceps, and an artificial vessel was anastomosed on the lateral wall (Fig. 2A). An arterial cannula was then introduced into the artificial vessel, and a right atrial cannula was inserted into the right atrial appendage. A left ventricular drainage tube was placed in the left atrial appendage, following which the cardiopulmonary bypass was established. The blood temperature was lowered to induce deep hypothermia, and the distal ascending aorta was obstructed. A histidine-tryptophan-ketoglutarate solution was then introduced through the left and right coronary arteries to induce cardiac arrest. The ascending aorta was transversally incised with circulatory arrest, and retrograde cerebral perfusion was performed via the superior vena cava. In accordance with preoperative CTA measurements and an intraoperative examination, a double-branched stent graft was appropriately selected and inserted into the aortic arch. The position and angle of the stent were adjusted using the delivery system's handle, and 2 branches were then introduced into the right and left IA, respectively (Fig. 2B). Next, the main and branch grafts were sequentially released through a four-stage deployment process. In the first 2 stages, the distal main graft and then the distal branch stent graft were released. After the position of the second branch stent graft was moderately adjusted, the surgeons fine-tuned the proximal main graft's position and the distance between the 2 branches. The proximal main graft was then released as the third stage of deployment, followed by the final stage, which involved deploying the proximal branch graft (Fig. 2C-2E). This sequence concluded with the withdrawal of the delivery system (Fig. 2F). The artificial vessel located proximal to the main stent was anastomosed with the ascending aorta (Fig. 2G). With rewarming, arterial blood flow was restored, and the heart automatically resumed its function (Fig. 2H). The anal temperature was subsequently rewarmed, the reduced volume was stopped and the CPB machine was gradually removed. Protamine was administered to neutralize the effects of heparin, the aortic cannula was removed and the side-connected artificial vessel was ligated, following which the procedure was deemed complete.

After the operation, the animals were administered penicillin to prevent infection. Additionally, postoperative anti-infection treatment was administered through intramuscular injection of cefoperazone/sulbactam sodium, and oral administration of aspirin was continued until the end of the experimental period.

### Follow-Up

The safety evaluation after the surgical procedure focused mainly on massive haemorrhage, stroke, paraplegia and stent thrombosis postoperatively; congestive heart failure, respiratory failure, aortic dissection and pyaemia were considered secondary indexes. In terms of feasibility evaluation, the primary index was the change in diameter of the artificial false lumen of the stent. The secondary evaluation indexes included the success rate of device delivery and release, the presence of endoleak and the displacement of the stent.

In this experimental study in animals, 4 follow-up time points were included, namely preoperative, intra-operative and follow-up end points. At each time point, the follow-up protocol involved recording the basic data for each animal, taking a complete blood count and assessing pathological and adverse events. CTA, digital subtraction angiography and blood sampling were carried out when the experimental end point was reached. Then the animals were euthanized and sampled after the completion of relevant examinations. Prior to euthanasia, heparin sodium was administered intravenously. Euthanasia was performed through intravenous injection of potassium chloride with the animals under deep anaesthesia. Postmortem, a skilled veterinarian conducted gross anatomical and histopathological evaluations of the heart, ascending aorta (including branched stent system), liver, spleen, lungs, kidneys and brain.

### Statistical analysis

Data were presented as means ± standard deviations. The paired *t*-test was utilized for differences in aortic diameter before the operation and at the end of the follow-up period in each group. SPSS 17 for Windows (IBM-SPSS, Armonk, NY, USA) was utilized for the statistical analysis. Results were categorized as statistically significant with the two-tailed *P*-level set at <0.05.

### Data presentation

All data necessary in Materials and Methods section are presented in Supplementary Table 1.

## RESULTS

In this experimental study, 12 animals were selected to receive branched stent implants in situ, comprising 6 in the 90-day group (group A) and 6 in the 180-day group (group B). Notably, all 12 animals with implants survived until the designated end point, with 6 animals from group A surviving 90 ± 30 days postoperatively and 6 animals from group B surviving 180 ± 30 days postoperatively. A summary of the basic information regarding the animals and the devices implanted in them is provided in Supplementary Table 2.

### Changes in perioperative characteristics of the animals

The 12 animals that were followed to the intended experimental end point exhibited perioperative characteristics according to the protocol, summarized in Supplementary Table 3. When the postoperative mean temperature, respiration, heart rate and blood pressure values in groups A and B were compared with the corresponding end-point values, it was observed that they exhibited a similar trend with no significant differences. However, weights in both groups differed significantly from the preoperative weight (group A follow-up: 114.77 ± 5.19 kg; preoperative: 91.75 ± 13.37 kg, *P *<* *0.05; group B: follow-up: 119.50 ± 1.50 kg, preoperation: 86.95 ± 9.06 kg, *P *<* *0.05). These findings indicated a similar surgical impact on the weight of the animals in both groups, with the survival time significantly affecting weights. Furthermore, the weights of the animals continued to increase after recovery from surgery.

### Analysis of complete blood count results

The present study reported the results of intraoperative total hemoglobin levels in 12 animals, as outlined in Supplementary Table 4. Following CPB, a significant difference was observed in the values of animals in group A compared to the preoperative results (after CPB: 7.78 ± 1.20 g/dl; preoperation: 9.68 ± 1.57 g/dl; *P *<* *0.05), whereas no significant difference was observed in group B. It was suggested that the observed changes in group A may be attributed to haemorrhage in individual animals and the CPB procedure.

### Stent graft performance

**Figure 3:**
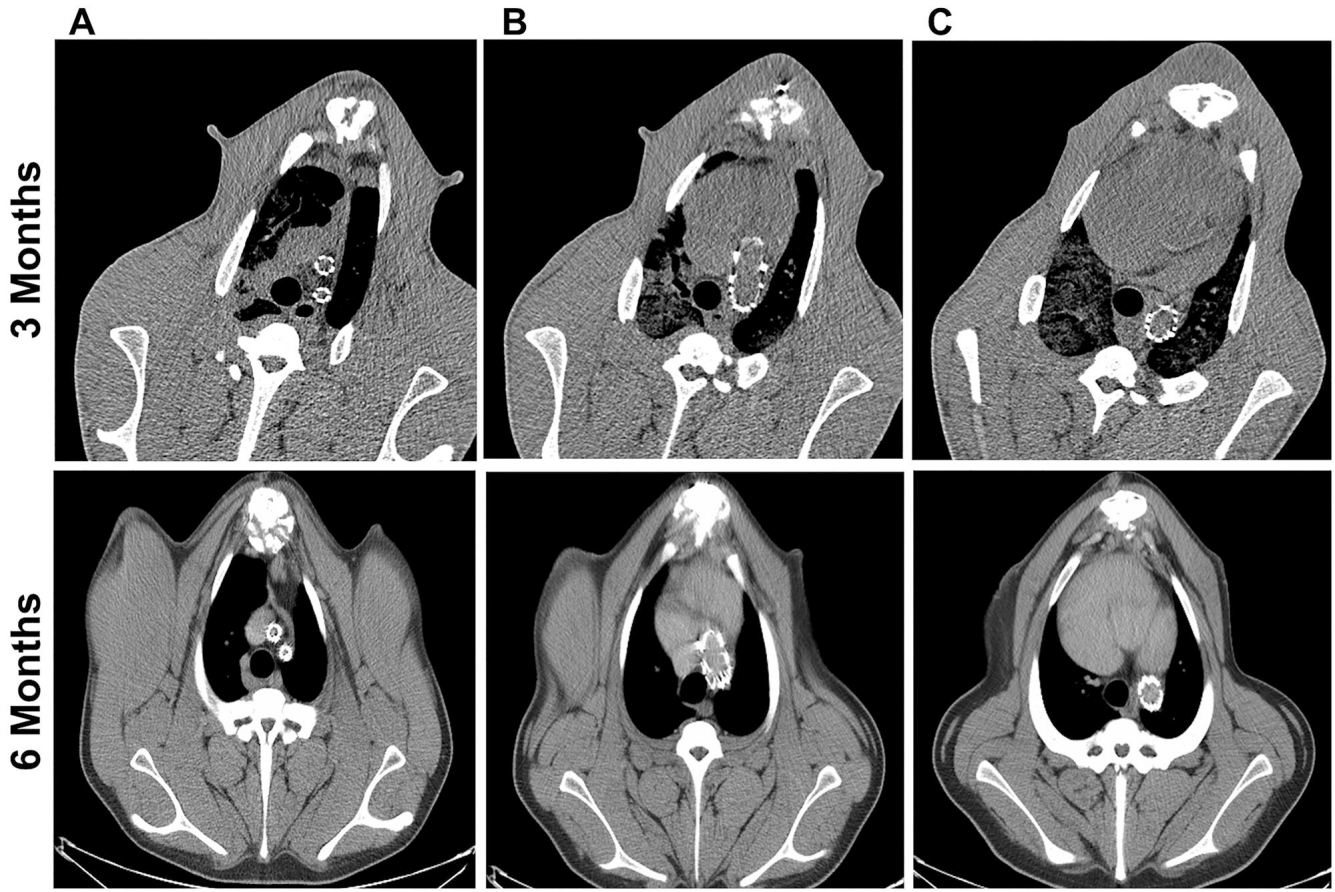
Computed tomography scans taken 90 and 180 days after implanting the double-branched stent graft system revealed no stent fracture or migration. (**A**) Brachiocephalic branch level; (**B**) arch level; (**C**) descending aortic level.

**Figure 4:**
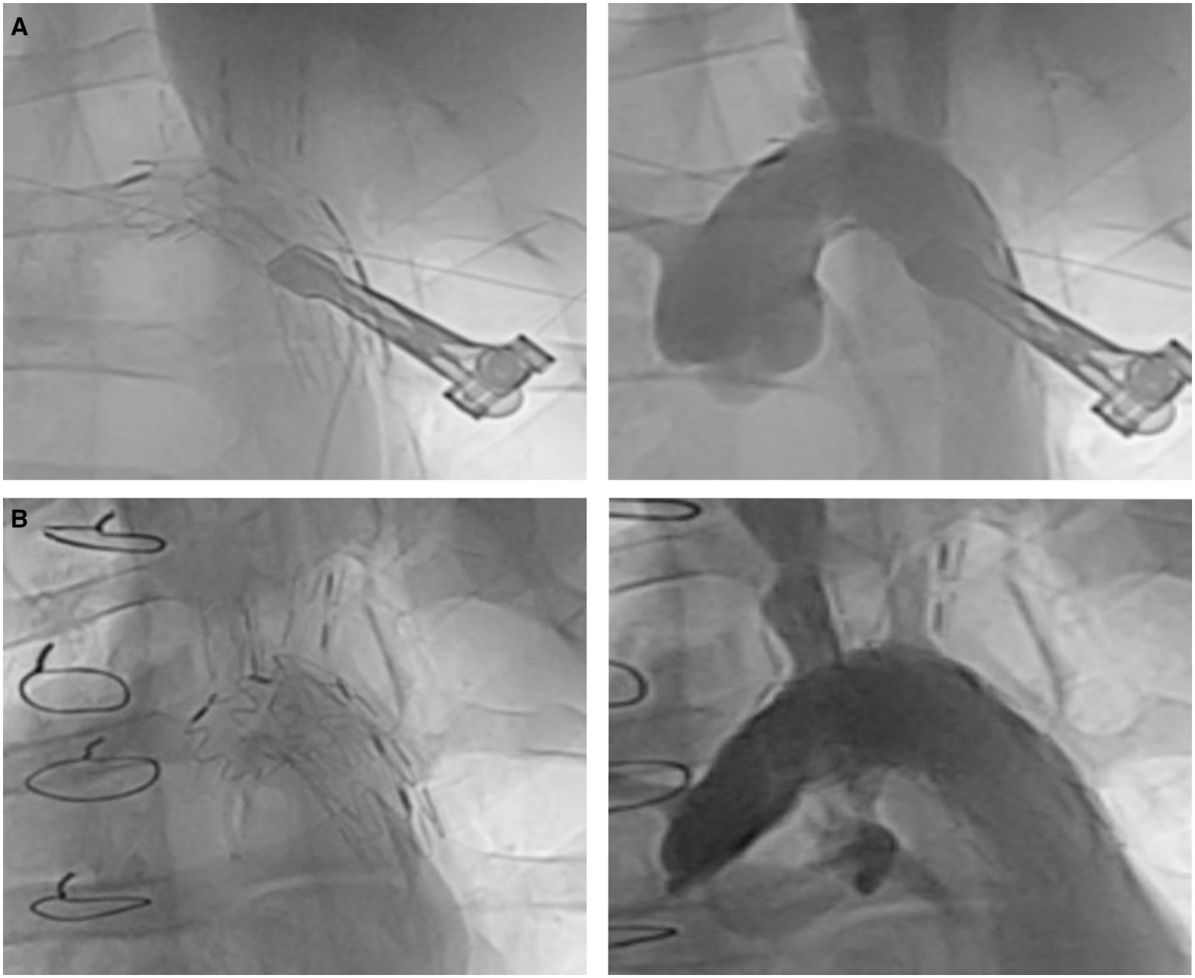
Digital subtraction angiography images showed that blood flow in the main stent and in the sidearm branches was unobstructed, without endoleak or dissection. (**A**) At 90 days follow-up; (**B**) at 180 days follow-up.

**Figure 5:**
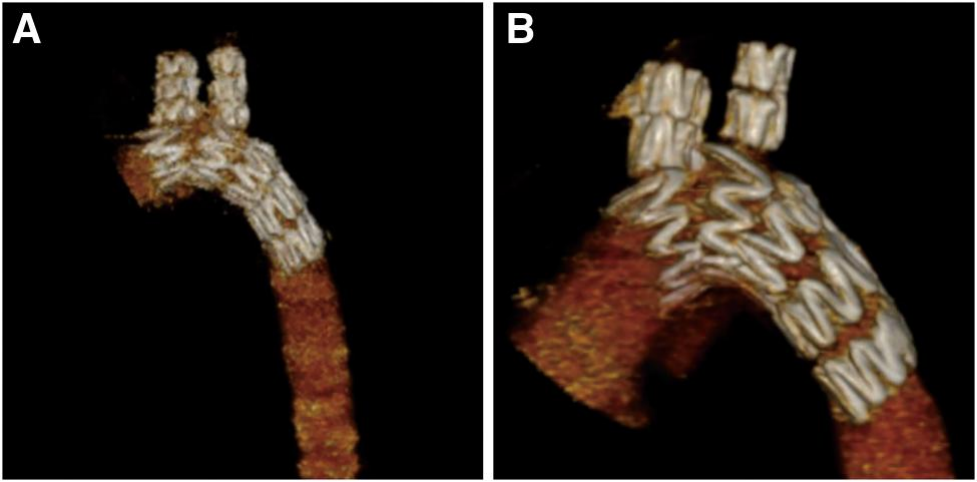
Three-dimensional computed tomography angiography images revealed no rupture, fracture, twist or migration in the main stent and in the sidearm branches. (**A**) At 90 days follow-up; (**B**) at 180 days follow-up.

Table 1 presents the imaging results of the 12 animals followed up to the expected experimental end-point. The follow-up examinations revealed no endoleak, no fracture and no stent migration (Figs. 3–5). Postoperative radial support of the stent graft proved reliable and was associated with the stent diameter. For diameters of 20 to 30 mm, the radial force exceeded 3 N. The mean diameter of the great vessels in group A was significantly different from the preoperative diameter, indicating a narrower mean aortic root diameter than existed preoperatively (*P *=* *0.042), whereas the mean diameter of the distal aortic arch was wider than it was preoperatively (*P *=* *0.031). However, the proximal diameter, distal diameter and the distance between double branches did not differ from those observed preoperatively. After comprehensive consideration, the observed differences in group A were attributable to the smaller diameter of the aortic opening of the stent graft in comparison to that of the actual aortic diameter (the material here was an artificial blood vessel), whereas the diameter of the distal aortic arch of the stent graft was larger than the actual aortic diameter (the material here was a stent). In group B, the mean diameter of the great vessels tended to be stable, and there was no significant difference compared to that before surgery. However, the mean diameter of the proximal branch and the spacing of the double branches were significantly different from those of the preoperative values, indicating that the mean diameter of the proximal branch was wider than that observed preoperatively (*P *=* *0.001), and the mean distance between the proximal and distal branches was wider than that observed preoperatively (*P *=* *0.010). Differences in group B likely resulted from the widening distance between the branches caused by the continuous development and growth of the animals. The principle of the increasing diameter of the proximal branch was similar to that of the distal aortic arch in group A (the material here was the stent).

**Table 1:** Imaging results of group A and group B

Group	Diameter (mm)	Preoperation	Follow-up		*P-*value
			90 ± 30 days 180 ± 30 days		
A	Aortic root	25.83 ± 2.14	23.67 ± 2.09*	/	0.042
	Proximal branch	13.73 ± 1.07	15.18 ± 0.89	/	0.148
	Distal branch	10.07 ± 2.02	11.32 ± 0.39	/	0.264
	Distal aortic arch	19.93 ± 2.74	22.00 ± 2.25*	/	0.031
	Distance between branches	5.88 ± 2.08	6.79 ± 0.99	/	0.342
B	Aortic root	28.32 ± 3.97	/	26.43 ± 2.90	0.155
	Proximal branch	13.60 ± 0.52	/	14.88 ± 0.50*	0.001
	Distal branch	9.95 ± 1.57	/	11.98 ± 0.86	0.067
	Distal aortic arch	23.07 ± 5.82	/	24.17 ± 3.06	0.521
	Distance between branches	5.84 ± 1.07	/	7.11 ± 1.01*	0.010

* Results were significantly different from those before the operation (*P *<* *0.05).

### Analysis of gross anatomical and histopathological examinations

**Figure 6:**
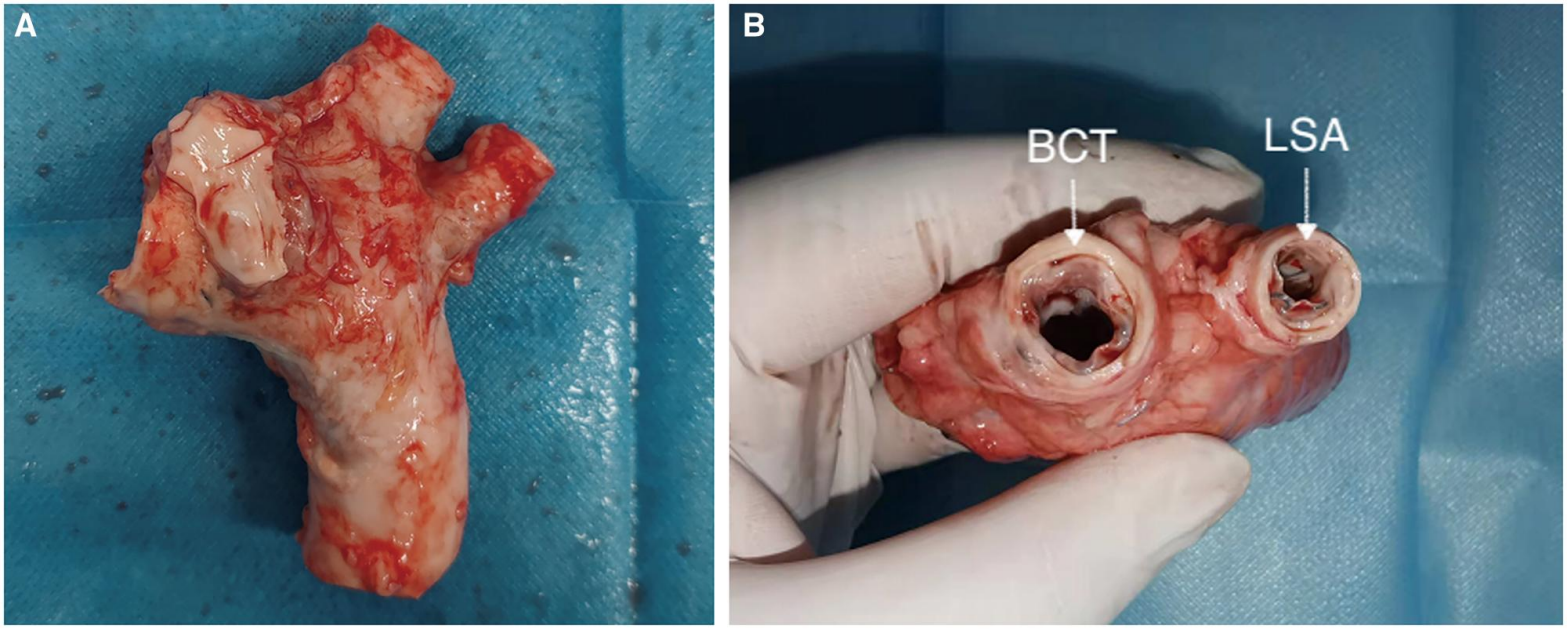
Gross anatomy of the aortic arch. (**A**) A representative animal necropsy showing the secure fixation and sealing of the stent graft; (**B**) macroscopic examination of the aortic arch axial section showing the brachiocephalic trunk and the brachiocephalic trunk.

**Figure 7:**
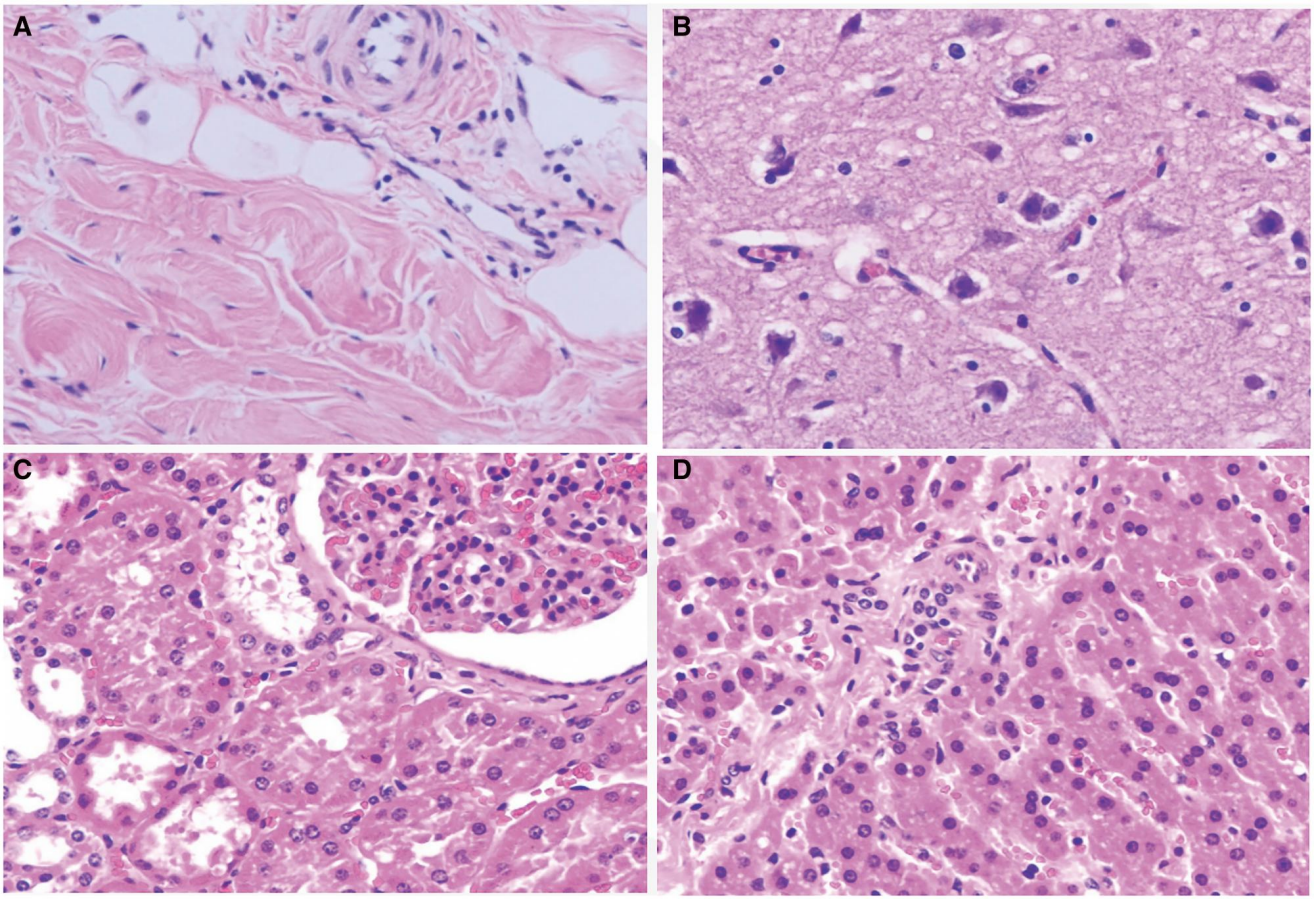
Hematoxylin and eosin staining images of the stent graft and various organs. (**A**) Normal vascular wall tissue was observed around the stent graft; (**B**) roughly normal cerebrum tissue; (**C**) roughly normal kidney tissue; and (**D**) roughly normal liver tissue.

Pathological examinations conducted on all animals revealed that branched stent grafts in both groups were successfully implanted in the aorta with no injuries, infections, dissections or stenosis observed in the ascending aorta, coronary artery, proximal and distal branches and the aortic arch. Furthermore, there were no intracardiac thromboses, and the stent grafts adhered securely to the vascular wall. Furthermore, none of the stent grafts was observed to be split, fractured, twisted or migrated, and no thrombus was formed on their surfaces (Fig. 6). Moreover, there were no observed infarctions on the surfaces of the lung, liver, spleen, kidney, cerebrum or cerebellum. In accordance with the relevant national standard (GB/T16886.6) for the biological evaluation of medical devices, part 6: Local reaction test after implantation (GB/T16886.6–2015/IS010993-6:2007), histological techniques were used to estimate the relative densities of the various types of cells and the integrity and thickness of fibrous sacs around the stent grafts implanted in the 12 animals. Endothelial cell layers covered the stent grafts, and the elastic layers of the medial wall remained intact (Fig. 7), indicating acceptable local tissue reaction and biocompatibility of the stent grafts.

## DISCUSSION

We developed and tested a novel double-branched stent graft system and four-stage deployment technology. Safety and feasibility were evaluated in a porcine model. With only stent implants in the proximal descending aorta, distal aortic arch LCCA and LSA, the system was designed to provide a simpler and safer alternative for aortic arch repair, the goal of which was to achieve the convenient manipulation of supra-arch vessels and simplify the procedure. Results indicated successful implementation of the double-branched stent graft in all study subjects throughout the follow-up period. Notably, there were no instances of endoleak, fracture or stent migration. Additionally, no gutter formation around the graft, stenosis or occlusion of sidearm branches was noted. All 12 subjects were free from significant complications. Besides, the delivery system demonstrated its ability to introduce the stent grafts to the target segments accurately and stably. It was successfully released and withdrawn from the access points without any technical difficulties.

Longitudinal dissection in type A acute aortic dissection often invades the arch, where careful choices of arch repair are required to balance the perioperative risks with the long-term benefits [15]. Hemi- and total arch replacements are still conventional and effective surgical techniques. Equipoise regarding those 2 strategies is limited by the concerns for reinterventions after conservative management and the complexity of an extensive operation [16, 17]. Sun’s procedure is widely used in China for performing extensive arch reconstruction, whereby incorporating the tetrafurcate vascular graft and the distal stent graft is a core advantage [18]. Nevertheless, Sun's procedure is difficult and time-consuming and could only done at major cardiovascular centres. The development of new stent graft systems with open technology for extensive arch repair has become a topic of interest in recent years. The triple-branched stent graft system for total arch repair proposed and developed by Chen *et al.* has achieved recognized excellence in clinical practice [19, 20], simplifying the repair procedure and reducing the duration of operations. However, 1 critical point is that their stent graft system has 3 sidearm stent grafts and a fixed distance between neighbouring branches, which makes optimal positions and directions of stents difficult to achieve. Other potential risks remain, such as misdirecting the graft into the false lumen, causing migration or occlusion or even malperfusion syndrome [21].

The double-branched stent graft system combines with the proximal artificial vessel, directing the stent graft into the descending aorta, aortic arch and 2 distal supra-arch vessels. This approach significantly reduces both CPB and the duration of the operation, accompanied by the simplified management of the supra-arch. Convenient arch limb repair protects the management from higher risks of operation-related complications. Given that the stent substitutes only for the left 2 sidearm arteries, both the proximal aortic arch and the IA are anastomosed with corresponding synthetic fabric following deployment, reducing the occurrence of endoleak.

Our double-branched device and deployment approach features several key highlights. The novel double-branched stent graft closely mimicked the anatomical structure of the arch. Implanting branch stents, which also avoided damaging the inner recurrent laryngeal nerve hidden between the LCCA and LSA, was more conducive to a safe operation [22]. Besides, product specifications are available in straight and tapered versions. The tapered design of the stent aids in mitigating excess wall stress in the distal stent region, thereby preventing the occurrence of stent graft-induced new entry. Conversely, straight-tube stents may offer a more satisfactory solution to the potential hazards of inadequate distal radial support and excessive rebound within the proximal landing zone in certain patient populations. Consequently, our device meticulously balances the complex interaction between the aortic taper and implant outcomes across diverse patients.

Branched stent grafts still face inevitable challenges, because continuous optimization of branch stent and supra-arch vessel matching remains necessary. Our stent graft system accommodates a wider range of aortic arch anatomical variations, particularly the distance between the LCCA and the LSA. Kondov *et al.* reported the morphology of the LSA as a justification for branched endovascular aortic arch repair, showing that the median distance between the LCCA offspring and the LSA offspring was 6.5 mm (4.0–11.0 mm) [23]. Implanting the branched stent graft with a fixed distance between the 2 supra-arch vessel grafts in current mainstream procedures could not be applied to best effect in most patients, because the distances between the 2 neighbouring sidearm vessels do not always match the available sizes of the current branched stent grafts. Based on the flexible design of our stent graft tube, surgeons could finely adjust the proximal main graft position, the distance between 2 branches and the directions of fixed rods towards branch vessels prior to the third stage of deployment. The distance between the 2 branched grafts can be adaptably varied from 4 mm to 12 mm, providing an optimal matching with the anatomically complex aortic arch. This flexibility in the distance between neighbouring branches, along with the precise navigation in the direction of the branch vessels, allows for the prevention of stent graft stenosis, occlusion or migration. Furthermore, it helps to avoid the complications associated with sidearm stent graft-induced new entry.

Additionally, our four-stage deployment technology made it a further refinement in wall apposition and deployment control. In the mainstream operating procedures using a stent graft for aortic repair, the fixed rod of the delivery system is pushed forwards to the aortic arch and the sidearm vessels through the arch or the ascending aortic incision, with or without the use of guidewires. Our delivery system ensures precise positioning of the stent graft. Current deployments involve the grafts releasing at the same time or sequentially [20, 24–26]. However, most studies favour a one-step release approach of the main stent graft. Complex landing zones and arch curvature challenge the graft conformance to the aortic arch geometry under the one-step deployment strategy. Because there is still a need for improved main tube graft deployment methods, our four-stage deployment technology was presented as a pioneer work in the existing literature. As mentioned, once the fixed rod delivering the stent graft was well-positioned, rather than releasing the stent graft completely at once, the distal stent was deployed first, followed by the first branch graft (in LSA). This step comprised the first 2 stages of deployment. After that, the attending surgeon could make fine proximal main graft position adjustments based on the length and curvature of the arch as well as on the angle of the orifice and the localization of the LCCA. Once the proximal main graft and the LCCA vascular stent were well positioned, they could be released sequentially, which comprised the third and fourth parts of the deployment. Based on our four-stage deployment technology, surgeons have more room to manoeuvre and a more optimized learning curve, better wall apposition and deployment control, thereby shrinking the false lumen and preventing type I/III endoleak.

Although the safety and feasibility of the stent grafts have shown promising results in animal studies, a significant challenge during clinical translation is how these stent grafts will adapt to the varied pathological conditions in clinical settings. Another animal experiment using canine models evaluated a double-branched stent graft, also designed for hybrid arch repair, in which postoperative imaging showed that both the main and the branched stent grafts were fully expanded and correctly positioned while 1 dog died of respiratory failure [27]. However, it must be acknowledged that the study comparability and clinical contributions of animal experiments are limited due to the small sample size and the wide heterogeneity of the animal models. Evaluating the safety, feasibility and efficacy of aortic stent grafts in clinical procedures remains the best practice guideline. We are a major collaborator with the randomized clinical trial, The Branch-based Intraoperative Stent System in the Treatment of Stanford A Aortic Dissection (BROAD), evaluating the double branched stent graft system and four-stage deployment technology. It was a prospective, multicentre, open and randomized controlled clinical trial. The study planned to enroll 257 participants from at least 10 institutions, randomly assigned to either Endovastec or PerMed's double-branched stent systems. Follow-ups were conducted at various intervals post-procedure for up to 2–5 years. The primary end point was all-cause mortality at 12 months, with a focus on non-inferiority comparison and long-term efficacy and safety assessments for device registration.

## LIMITATIONS

There are various limitations to our preclinical study that should be highlighted. To begin, it should be acknowledged that the anatomical components of the porcine model utilized in this study were only marginally similar to human anatomical components. Therefore, the findings may not entirely match those obtained in human pathological conditions. Moreover, the pigs in our study were healthy, without aortic dissection or aortic aneurysm, which cannot precisely mimic the pathological circumstances of aortic emergencies. Finally, the durability of this double-branched stent graft in the aortic arch remains to be fully elucidated. As with any innovative medical device, further long-term follow-up studies are warranted to assess the potential for adverse events and to optimize the clinical performance of this promising device.

## CONCLUSIONS

In summary, the innovative double-branched stent graft system is a reliable and feasible option for aortic arch repair in accordance with our preclinical evaluation with porcine models. Four-stage deployment technology has further enhanced the safety and efficacy by optimizing graft release.

## Supplementary Material

ivae049_Supplementary_Data

## Data Availability

The data underlying this article will be shared on reasonable request to the corresponding author.

## ABBREVIATIONS

CPB: Cardiopulmonary bypass
CTA: Computed tomography angiography
IA: Innominate artery
LCCA: Left common carotid artery
LSA: Left subclavian artery
