# Supplementary material for: Double-branched stent graft and four-stage deployment in total arch repair: safety and feasibility evaluation in porcine models
Source: Interdiscip Cardiovasc Thorac Surg. 2024 Mar 16;38(4):ivae049. doi: 10.1093/icvts/ivae049 (PMC11014789; doi:10.1093/icvts/ivae049)
Supplement: ivae049_Supplementary_Data [file ivae049_supplementary_data.zip › Revised Manuscript R1-Double-branched stent graft - marked.docx]

**Double-branched stent graft and four-stage deployment in total arch repair: safety and feasibility evaluation in porcine models**

Chenhao Wang^1^*; Wenfan Li^1^*; Peng Yang^1^; Chen Lu^1^; Yu Zhang^1^; Haiyue Wang^1^; Zhenghua Xiao^1^; Jia Hu^1,2#^

1. Department of Cardiovascular Surgery, West China Hospital, Sichuan University, Chengdu, Sichuan Province, P.R. China

2. Department of Cardiothoracic Surgery, West China Guang’an Hospital, Sichuan University, Guang’an, Sichuan Province, P.R. China

*These authors contributed equally to the study.

| #Corresponding to: | Jia Hu |
| --- | --- |
|  | 1. Department of Cardiovascular Surgery, West China Hospital,  Sichuan University, Chengdu, People’s Republic of China |
|  | Guo Xue Alley 37, Chengdu, Sichuan, People’s Republic of China, 610041  2. Department of Cardiothoracic Surgery, West China Guang’an Hospital, Sichuan University, Guang’an, Sichuan Province, P.R. China  No.1, Section 4, Binhe Road, Guang’an, Sichuan, People’s Republic of China, 638500 |
|  | Tel: +86 028 85421833 |
|  | Fax: +86 028 85421833  E-mail: jiahu@wchscu.edu.cn |

Word Count: 4162

**Abstract and Keywords**

**Objectives**: The primary objective of this research was to evaluate the safety and feasibility of an innovative double-branched stent graft system employing four-stage deployment technology for aortic arch repair in porcine models.

**Methods:** The double-branched stent graft system consisted of a proximal polyester artificial blood vessel, the main and double-branched stent grafts, and a delivery system. We utilized 12 healthy pigs as experimental animals (6 per group) in this study. Post-implantation, samples were collected at 90 and 180 days of follow-up. Preoperative, postoperative imaging, and intraoperative arterial blood gas analyses were performed. After euthanasia, the implanted product, surrounding tissue, and major organs were collected for pathological analysis.

**Results:** The technical success rate of stent graft implantation was 100% (12/12). All animals survived to the experimental endpoint. Perioperative assessments showed intact stent grafts, and imaging features at the end of the follow-up revealed neither endoleak nor device migration. No major adverse cardiovascular events were observed in the postoperative follow-up. Pathological examination confirmed satisfactory biocompatibility of the stent graft.

**Conclusion:** This innovative double-branched stent graft system with four-stage deployment technology was affirmed as a safe and feasible option for aortic arch repair in accordance with our preclinical evaluation with porcine models.

**Keywords**: Aortic arch repair; Double-branched stent graft; Preclinical study; Porcine; Type A acute aortic dissection.

**Introduction**

Aortic diseases, particularly Type A acute aortic dissection (TAAAD), are recognized as life-threatening cardiovascular emergencies. Hemi-arch replacement is a reliable option, while associated with reduced occurrence of false lumen thrombosis and higher incidence of reintervention [1]. Total arch replacement enhances survival and minimizes redo-surgeries, yet it demands significant surgical expertise for delicate grafting and potentials risks due to sternotomy, cardiopulmonary bypass (CPB), deep hypothermic circulatory arrest, and prolonged selective cerebral perfusion [2-5].

Advanced arch repair endeavors to combine the benefits of conventional surgery and endovascular treatment [6-8]. The Thoraflex Hybrid graft and the E-vita Open Plus are the two most widely utilized frozen elephant trunk components of hybrid arch repairs [9,10]. The mainstream frozen elephant trunks, however, are associated with their own set of challenges, such as type I and type II endoleak and distal dissections [11,12]. Notably, triple branched stent grafts have proved to be a viable therapeutic choice for complete arch repair in recent years [13,14]. However, the anatomically intricate aortic arch mandates the implantation of various-sized three-branch stents, posing a limitation to the clinical utility of surgical procedures.

We have recently developed a double-branched stent graft system and four-stage deployment technology, indicated for comprehensive arch repair under open replacement. Total arch repair can be gracefully accomplished by deploying the stent graft into the proximal descending aorta, the left subclavian artery (LSA), the aortic arch, and the left common carotid artery (LCCA), also enabling straightforward execution of proximal and distal vascular anastomoses. Flexible distance between two distal neighboring branches of our device allows adaptation to a wider range of patients, and the four-stage deployment technology ensures improved wall apposition and deployment control. Collectively, these features could enhance the outcomes by reducing complications after stent graft implantation. In this article, we reported the early results from our preclinical evaluation involving porcine models implanted with this advanced stent graft and four-stage deployment technology.

**Materials and Methods**

**Study design**

The present study was a preclinical in vivo animal research that involved implanting aortic stent grafts in animals to assess the device's safety and feasibility. All experimental procedures strictly followed the ARRIVE (Animal Research: Reporting of In Vivo Experiments) guidelines. Furthermore, the study complied with ethical principles for animal experimentation and was approved by the animal experimental ethics review committee (Ethics Approval Number: SS-2020-PHZJ).

**The novel double-branched stent graft and the delivery system**

Conceived and fabricated by Permed Biomedical Engineering Co., LTD (Beijing, China), this double-branched stent graft system comprised of a proximal polyester artificial blood vessel, the main and double-branched stent graft, and a corresponding delivery system, illustrated in **Figure 1**. The supporting body of the covered stent was manufactured using Ni-ti alloy wire with shape memory properties. The "W" shape structure of the wire rendered the stent both compressible and self-expanding. The main stent graft consisted of the proximal section for effective arch repair, and the distal section acting as the stented elephant trunk. The diameter of the proximal and distal stents, branch length and diameter, and distal stent length are designed to be variable. Furthermore, the main graft between the LSA and the LCCA was suitably soft and thin, without alloy wires for support, which allows for transverse folding to facilitate its placement and adjustment during surgical procedures. Both the inner and outer layers of the bare stent were coated with a polytetrafluoroethylene membrane, which exhibited effective blood-blocking properties and had excellent biocompatibility. Proximal and distal ends of the stent were covered with a polyester cloth to promote sealing after implantation and prevent endoleak. Additionally, a proximal artificial vascular fabric, sutured adjacent to the covered stent, was designed to aid in reducing the risk of postoperative hemorrhage. The delivery system comprised handle parts and release knobs. After pre-installing the stent in the stent-loading area on the delivery device, the stent was sent to the target location by pulling and releasing the cable. The stent automatically sprang open, following which the delivery device was removed. During surgery, the stent was delivered to the target site via the fixed rod after opening the aorta. The stent created an artificial false lumen at the site of injury, isolating the high-pressure blood flow from the lesion site and preventing blood pressure-induced damage.

**Animal Preparation**

To ensure anatomical and pathological similarity with humans and hemodynamic similarity, we utilized Chinese hybrid Landrace porcine models. As per the draft Guiding Principles for Technical Review of Animal Experimental Studies of Medical Devices from the Medical Device Technical Review Center of the National Medical Products Administration of the People's Republic of China, twelve pigs were employed for this study, randomly divided into Group A and Group B. The animals were individually marked with ear tags for identification. The experimental facilities and animals were provided by an animal experiment institute (Yinsnake Medical Technology Co., LTD, Guangzhou, China) accredited by the Standardization Administration of China for Laboratory animal - Requirements of environment and housing facilities. Prior to the surgery, the animals were acclimatized in the feeding room. After the implantation, animals were transferred to the care unit for monitoring and later transferred back to the feeding room once recovered. Each animal was kept in an individual cage and had access to food and water before and after the surgery. Water was freely accessible, while the feeding based on individual weight was quantitative, administered twice daily at fix times. The animal facility room temperature and relative humidity were suitable.

The animals underwent fasting and restricted water intake prior to the initiation of anesthesia. Prior to skin preparation, Sutaride (VIRBAC, Paris, French) and atropine sulfate were administered intramuscularly to provide sedation. After successful induction of anesthesia with propofol (Guangdong Jiabo Pharmaceutical, Qingyuan, China), endotracheal intubation was performed, and anesthesia was maintained using propofol and isoflurane. Mechanical ventilation was employed to assist breathing, and intravenous access was established. Additionally, heart rate and blood pressure monitoring, as well as blood gas analysis, were performed.

**Operation Procedures and Deployment Technology**

The device size selection was guided by preoperative computed tomography angiography (CTA). Following a routine sternotomy, the proximal portion of the right innominate artery (IA) was meticulously isolated from the surrounding tissue. The activated clotting time of the whole blood was measured to ensure adequate heparinization. The right IA was clamped with lateral forceps, and an artificial vessel was anastomosed on the lateral wall (**Fig. 2A**). Arterial cannula was then introduced into the artificial vessel, and a right atrial cannula was inserted into the right atrial appendage. A left ventricular drainage tube was placed in the left atrial appendage, following which the cardiopulmonary bypass was established. The blood temperature was lowered to induce deep hypothermia, and the distal ascending aorta was obstructed. Histidine-tryptophan-ketoglutarate solution was then introduced through the left and right coronary to induce cardiac arrest. The ascending aorta was transversally incised with circulatory arrest, and retrograde cerebral perfusion was performed via the superior vena cava. In accordance with preoperative CTA measurements and intraoperative examination, a double-branched stent graft was appropriately selected and inserted into the aortic arch. The position and angle of the stent were adjusted using the delivery system's handle, and two branches were then introduced into the right and left IA, respectively (**Fig. 2B**). Next, the main and branch grafts were sequentially released through a four-stage deployment process. In the first two stages, the distal main graft and then the distal branch stent graft were released. After the position of the second branch stent graft was moderately adjusted, surgeons fine-tuned the proximal main graft's position and the distance between the two branches. The proximal main graft was then released as the third stage of deployment, followed by the final stage, which involved deploying the proximal branch graft (**Fig. 2C-2E**). This sequence concluded with the withdrawal of the delivery system (**Fig. 2F**). The artificial vessel located proximal to the main stent was anastomosed with the ascending aorta (**Fig. 2G**). With rewarming, arterial blood flow was restored, and the heart automatically resumed its function (**Fig. 2H**). The anal temperature was subsequently rewarmed, the reduced volume was stopped, and the CPB machine was gradually removed. Protamine was administered to neutralize the effects of heparin, the aortic cannula was removed and the side-connected artificial vessel was ligated, following which the procedure was deemed complete.

After the operation, the animals were administered U penicillin to prevent infection. Additionally, postoperative anti-infection treatment was administered through intramuscular injection of cefoperazone/sulbactam sodium, and oral administration of aspirin was continued until the end of the experimental period.

**Follow-Up**

The safety evaluation after the surgical procedure mainly focused on massive hemorrhage, stroke, paraplegia, and stent thrombosis postoperatively, while congestive heart failure, respiratory failure, aortic dissection, and pyaemia were considered secondary indexes. In terms of feasibility evaluation, the primary index was the change in diameter of the artificial false lumen of the stent, while the secondary evaluation indexes included the success rate of device delivery and release, the presence of endoleak, and the displacement of the stent.

In this animal experimental study, four follow-up time points were included, namely pre-operation, intra-operation, and follow-up endpoint. At each time point, the follow-up protocol involved recording the basic animal information, complete blood count (CBC), and assessing pathological and adverse events. CTA, digital subtraction angiography, and blood sampling were carried out when the experimental endpoint was reached, and then animals were euthanized and sampled after the completion of relevant examinations. Prior to euthanasia, heparin sodium was administered intravenously. Euthanasia was performed under deep anesthesia through intravenous injection of potassium chloride. Postmortem, a skilled veterinarian conducted gross anatomic and histopathologic evaluations of the heart, ascending aorta (including branched stent system), liver, spleen, lungs, kidneys, and brain.

**Statistical Analysis**

Data were presented as means ± standard deviations. Paired t-test was utilized for differences in aortic diameter before surgery and at the end of follow-up in each group. SPSS 17 for Windows was utilized for the statistical analysis. Results were categorized as statistically significant with the two-tailed *p*-level set at <0.05.

**Data presentation**

All data necessary in Materials and Methods section was presented in the **Supplementary Table 1**.

**Results**

In this experimental study, 12 animals were selected for branched stent implantation in situ, comprising 6 in the 90-day (Group A) and 6 in the 180-day group (Group B). Notably, all 12 implanted animals survived until the designated endpoint, with 6 animals from Group A surviving 90±30 days post-operation, and 6 animals from Group B surviving 180±30 days post-operation. A summary of the basic information regarding the animals and the devices implanted in them is provided in **Supplementary** **Table 2**.

**Changes in perioperative characteristics about animals**

The 12 animals that were followed up to the intended experimental endpoint underwent perioperative characteristics according to the protocol, summarized in **Supplementary** **Table 3**. Mean temperature, respiration, heart rate, and blood pressure values before surgery in Groups A and B were compared with the corresponding endpoint value, and it was observed that they exhibited a similar trend of change with no significant difference. However, weights in both groups significantly differed from the preoperative weight (Group A: Follow-up: 114.77±5.19 Kg, Preoperation: 91.75±13.37 Kg, *p* < 0.05; Group B: Follow-up: 119.50±1.50 Kg, Preoperation: 86.95±9.06 Kg, *p* < 0.05). These findings indicated a similar surgical impact on the weight of animals in both groups, with the survival time significantly affecting weights. Furthermore, the weight of animals continued to increase after recovery from surgery.

**Analysis of** [**complete blood count**](http://www.baidu.com/link?url=Uxh8AcGVRO4Dh4OPt7VV-AdfelsW8foakiGYmAJyw5_x0iUMPS1XQ4z-clv8FbgLfmUo-vlKFNUFoQlYFFi2VcKswSOwSYCLGUOQ8so6aptf9zuVJCQ8-s5Rnc3obh_L) **results**

The present study reported the results of intraoperative total hemoglobin in 12 animals, as outlined in **Supplementary** **Table 4**. Following CPB, a significant difference was observed in values of animals in Group A compared to the preoperative results (After CPB: 7.78±1.20 g/dl; Preoperation: 9.68±1.57 g/dl; *p* < 0.05), whereas no significant difference was observed in Group B. It was suggested that the observed changes in Group A may be attributed to individual animal hemorrhage and the CPB procedure.

**Stent graft performance**

**Table 1** presented the imaging results of the 12 animals followed up to the expected experimental endpoint. The follow-up revealed no endoleak, no fracture, and no stent migration (**Figure 3-5**). Postoperative radial support of the stent graft proved reliable, associated with the stent diameter. For diameters of 20-30mm, radial force exceeds 3N. The mean diameter of great vessels in Group A was significantly different from preoperation, indicating a narrower mean aortic root diameter than preoperation (*p* = 0.042), while the mean diameter of the distal aortic arch was wider than preoperation (*p* = 0.031). However, the proximal diameter, distal diameter, and the distance between double branches did not differ from preoperation. After comprehensive consideration, the observed differences in Group A were attributable to the smaller diameter of the aortic opening of the stent-graft than the actual aortic diameter (the material here was an artificial blood vessel), while the diameter of the distal aortic arch of the stent-graft was larger than the actual aortic diameter (the material here was a stent). In Group B, the mean diameter of great vessels tended to be stable, and there was no significant difference compared to that before surgery. However, the mean diameter of the proximal branch and the spacing of double branches were significantly different from preoperation, indicating that the mean diameter of the proximal branch was wider than preoperation (*p* = 0.001) and the mean distance between the proximal and distal branches was wider than preoperation (*p* = 0.010). Differences in Group B likely resulted from the widening distance between the branches caused by the continuous development and growth of animals. The principle of the increasing diameter of the proximal branch was similar to that of the distal aortic arch in Group A (the material here was the stent).

**Analysis of Gross Anatomic and Histopathologic Examinations**

Pathological examination conducted on all animals revealed that branched stent grafts in both groups were successfully implanted in the aorta with no injuries, infections, dissection, or stenosis observed in the ascending aorta, coronary artery, proximal and distal branches, and aortic arch. Furthermore, there were no intracardiac thromboses, and the stent grafts adhered securely to the vascular wall. Furthermore, none of the stent grafts were observed to be split, fractured, twisted, or migrated, and no thrombus was formed on their surface (**Figure 6**). Moreover, there were no observed infarctions on the surfaces of the lung, liver, spleen, kidney, cerebrum, or cerebellum. In accordance with the relevant national standard (GB/T16886.6) on the biological evaluation of medical devices-part 6: local reaction test after implantation (GB/T16886.6-2015/IS010993-6:2007), histological techniques were employed to estimate the relative density of various types of cells and the integrity and thickness of fibrous sacs around the stent grafts implanted in the 12 animals. Endothelial cell layers covered the stent grafts, and the elastic layers of the medial wall remained intact (**Figure 7**), indicating acceptable local tissue reaction and biocompatibility of the stent grafts.

**Discussion**

In this study, we developed and tested a novel double-branched stent-graft system and four-stage deployment technology. Safety and feasibility were evaluated in porcine model. With only stent implantations in the proximal descending aorta, distal aortic arch LCCA, and LSA, the system was designed to provide a simpler and safer alternative for aortic arch repair, which aims to achieve the convenient manipulation of supra-arch vessels and simplifier procedure. Results indicated successful implementation of the double-branched stent graft in all study subjects throughout the follow-up period. Notably, there were no instances of endoleak, fracture, or stent migration. Additionally, no gutter formation around the graft, stenosis, or occlusion of sidearm branches was noted. All 12 subjects were free from significant complications. Besides, the delivery system demonstrated its ability to introduce the stent grafts to the target segments accurately and stably. It was successfully released and withdrawn from the access points without any technical difficulties.

Longitudinal dissection in TAAAD often invades the arch, where careful choices of arch repair is required to balance the perioperative risks with the long-term benefits [15]. Hemi and total arch replacement are still conventional and effective surgical techniques. Equipoise regarding those two strategies is limited by the concerns for reinterventions after conservative management and the complexity of extensive operation [16,17]. Sun’s procedure is widely used in China for performing extensive arch reconstruction with incorporating the tetrafurcate vascular graft and distal stent graft as core advantages [18]. Nevertheless, Sun's procedure, is difficult and time-consuming, could only done at major cardiovascular centers. Developments of new stent graft systems with open technology for extensive arch repair comes as a topic of interest in recent years. Triple-branched stent graft system for total arch repair proposed and developed by Chen et al. has achieved recognized excellence in clinical practice [19,20], simplifying the repair procedure and reducing the duration of operations. However, one critical point is that their stent graft system owns three sidearm stent grafts and fixed distance between neighboring branches, which makes optimal positions and directions of stents difficult to achieve. Other potential risks remain, such as misleading the graft into the false lumen, causing migration or occlusion, even malperfusion syndrome [21].

Double-branched stent graft system combines with the proximal artificial vessel, developing the stent graft into the descending aorta, aortic arch, and two distal supra-arch vessels. This approach significantly reduces both CPB and operation duration, accompanied by the simplified management of supra-arch. Convenient arch limb repair protects the management from higher risks of operation-related complications. Given that the stent substitutes only the left two sidearm arteries, both the proximal aortic arch and the IA are anastomosed with corresponding synthetic fabric following deployment, reducing the occurrence of endoleak.

Our double-branched device and deployment approach features several key highlights. The novel double-branched stent graft closely mimicsed the arch's anatomical structure. Implanting branch stents also avoided damaging the inner recurrent laryngeal nerve hidden between the LCCA and LSA, more conducive to the surgery safety [22]. Besides, product specifications are available in straight and tapered versions. The tapered design of the stent aids in mitigating excess wall stress in the distal stent region, thereby preventing the occurrence of stent graft-induced new entry. Conversely, straight-tube stents may offer a more satisfactory solution to the potential hazards of inadequate distal radial support and excessive rebound within the proximal landing zone in certain patient populations. Consequently, our device meticulously balances the complex interaction between aortic taper and implantation outcomes across diverse patients.

Branched stent grafts still face inevitable challenges, as continuous optimization of branch stent and supra-arch vessel matching remains necessary. Our stent graft system accommodate a wider range of aortic arch anatomical variations, particularly the distance between the LCCA and LSA. Stoyan Kondov et al. reported the morphology of the LSA as implications for branched endovascular aortic arch repair, showing that the median distance between the LCCA offspring and the LSA offspring was 6.5 mm (4.0-11.0 mm) [23]. An implantation of the branched stent graft with a fixed distance between the two supra-arch vessel graft in current mainstream could not be applied to best effect in most patients, because the distances between two neighboring sidearm vessels do not always match the available sizes of the current branched stent grafts. Based on the flexible design of our stent graft tube, surgeons could finely adjust the proximal main graft position, distance between two branches, and the directions of fixed rods towards branch vessels prior to the third stage of deployment. The distance between the two branched graft can be adaptably varied from 4mm to 12mm, providing an optimal matching with the anatomically complex aortic arch. This flexibility in the distance between neighboring branches, along with the precise navigation in the direction of branch vessels, allows for the prevention of stent graft stenosis, occlusion, or migration. Furthermore, it helps to avoid the complications associated with sidearm stent graft-induced new entry.

Additionally, our four-stage deployment technology made it a further refinement in wall apposition and deployment control. In the mainstream operation procedures with stent graft for aortic repair, the fixed rod of the delivery system is pushed forwards to the aortic arch and sidearm vessels through the arch or ascending aortic incision, with or without guiding of guidewires. Our delivery system ensures precise stent graft positioning. Current deployments involve the grafts releasing at the same time or sequentially [20,24-26]. However, most studies favor a one-step release approach of the main stent graft. Complex landing zones and arch curvature post challenges to the graft conformance to the aortic arch geometry under the one-step deployment strategy. There remains a need for improved main tube graft deployment methods and our four-stage deployment technology was presented as a pioneer work in existing literature. As mentioned, once the fixed rod delivering stent graft was well-positioned, rather than releasing stent graft completely at once, the distal stent was deployed first, followed by the first branch graft (in LSA). This was the first two stages of deployment. After that, attending surgeon could make fine proximal main graft position adjustments based on the length and curvature of the arch, as well as the angle of orifice and localization of the LCCA. Once the proximal main graft and the LCCA vascular stent were well positioned, they could be sequentially released, and this was the third and fourth part of the deployment. Based on our four-stage deployment technology, surgeons have more room to maneuver and a more optimized learning curve, features better wall apposition and deployment control, shrinking the false lumen and preventing type I/III endoleak.

While the stent grafts' safety and feasibility have shown promising results in animal studies, a significant challenge during clinical translation is how these stent grafts will adapt to the varied pathologic conditions in clinical settings. Another animal experiment using canine models evaluated a double-branched stent graft also for hybrid arch repair, in which postoperative imaging showed that both the main and branched stent-graft were fully expanded and correctly positioned while one dog died of respiratory failure [27]. However, it must be acknowledged that the study comparability and clinical contribution of animal experimentals are limited due to the small sample size and strong heterogeneity of the animal models. Evaluating the safety, feasibility, and efficacy of aortic stent grafts in clinical procedures remains the best practice guideline. We come as a major collaborator of the RCT: The Branch-based Intraoperative Stent System in the Treatment of Stanford A Aortic Dissection (“BROAD”), evaluating the double branched stent graft system and four-stage deployment technology. It was a prospective, muticenter, open, and randomized controlled clinical trial. The study planned to enroll 257 participants from at least 10 institutions, randomly assigned to either Endovastec or PerMed's double-branched stent systems. Follow-ups were conducted at various intervals post-procedure, up to 2-5 years. The primary endpoint was all-cause mortality at 12 months, with a focus on non-inferiority comparison and long-term efficacy and safety assessments for device registration.

**Limitations**

There are various limitations to our preclinical study that should be highlighted. To begin, it should be acknowledged that the porcine model utilized in this study only provided a limited similarity of human anatomical components, and therefore, the findings may not be entirely match with the human pathology. Moreover, pigs in our study were healthy models without aortic dissection or aortic aneurysm, which cannot precisely mimic the pathological circumstances of aortic emergencies. Finally, the durability of this double-branched stent graft in the aortic arch remains to be fully elucidated. As with any innovative medical device, further long-term follow-up studies are warranted to assess the potential for adverse events and to optimize the clinical performance of this promising device.

**Conclusions**

In summary, the innovative double-branched stent graft system is a reliable and feasible option for aortic arch repair in accordance with our preclinical evaluation with porcine models. Four stage deployment technology has further enhanced the safety and efficacy by optimizing graft-release.

**Funding:** This work was provided by the National Natural Science Foundation of China [81670327, 81300155], Sichuan Science and Technology Program [2019YJ0046, 2022YFS0358], 1·3·5 project for disciplines of excellence, West China Hospital, Sichuan University [2020HXJS015], 1·3·5 project for disciplines of excellence–Clinical Research Incubation Project, West China Hospital, Sichuan University [2019HXFH027, 2020HXFH043] and Project of China International Medical Foundation [Z-2016-23-2101-27].

**Conflict of interest:** none declared.

**Data availability statements**: The data underlying this article will be shared on reasonable request to the corresponding author.

**Author contributions:**

**Chenhao Wang**-Data curation; Formal analysis; Investigation; Software; Writing -

original draft; Writing - review & editing.

**Wenfan Li**-Data curation; Formal analysis; Investigation; Validation; Writing - original draft.

**Peng Yang**-Data curation; Investigation; Software; Validation; Writing - review & editing.

**Chen Lu**-Formal analysis; Investigation; Software; Visualization.

**Yu Zhang**-Formal analysis; Software; Validation.

**Haiyue Wang**-Software; Validation; Visualization.

**Zhenghua Xiao**-Conceptualization; Methodology; Supervision.

**Jia Hu**-Conceptualization; Methodology; Funding acquisition; Project administration; Resources; Supervision; Validation.

**Figure Captions**

**Graphical Abstract**: Introduction of the stent graft and implantation procedure, and primary outcomes of safety and feasibility evaluation. *, *p* < 0.05; ^✝^, including massive hemorrhage, stroke, paraplegia, and stent thrombosis. .

**Figure 1**: The novel double-branched stent graft system. (**A**) The stent graft is composed of a proximal polyester vascular graft fabric, a distal Ni-ti alloy wire bare stent, and a polytetrafluoroethylene membrane. The stent comprised a main graft and two branched grafts. (**B**, **C**) The delivery system to match the newly designed double-branched stent graft. AA, aortic arch; LSA, left subclavian artery; LCCA, left common carotid artery.

**Figure 2**: Diagrams of implantation procedures for the double-branched stent graft and operation. (**A**) The right innominate artery was clamped and anastomosed laterally as an artificial vessel. (**B**) The double-branched stent graft was placed into the aortic arch. (**C**) The distal main graft and then the distal branch graft were released as the first two stages of deployment. (**D**) The position of the second branch stent graft in the right innominate artery was adjusted moderately. (**E**) The proximal main graft and theproximal branch graft were released as the second deployment. (**F**) The delivery system was withdrawn from the aorta. (**G**) The artificial vessel located proximal to the main stent was anastomosed with the ascending aorta. (**H**) The artery blood flow was restored after anastomosing.

**Figure 3**: CT of 90 and 180 days after the implantation of the double-branched stent-graft system revealed no stent fracture and migration: (**A**) Brachiocephalic branch level, (**B**) Arch level, (**C**) Descending aortic level.

**Figure 4**: The digital subtraction angiography (DSA) showed that blood flow in the main stent and the sidearm branches were unobstructed, without endoleak or dissection: (**A**) 90 days of follow-up, (**B**) 180 days of follow-up.

**Figure 5**: The 3-dimensional computed tomography angiography (CTA) images revealed no rupture, fracture, twist, or migration in the main stent and the sidearm branches: (**A**) 90 days of follow-up, (**B**) 180 days of follow-up.

**Figure 6**: Gross anatomic of Aortic arch: (**A**) A representative animal necropsy showing the secure fixation and sealing of the stent graft. (**B**) Macroscopic examination of the aortic arch axial section showing BCT and LSA.

**Figure 7**: Hematoxylin and eosin (H&E) staining images of the stent graft and various organs: (**A**) Normal vascular wall tissue was observed around the stent graft, (**B**) roughly normal cerebrum tissue, (**C**) roughly normal kidney tissue, and (**D**) roughly normal liver tissue.

**Table Caption**

Table 1. Imaging Results of Group A and Group B.

| Group | Diameter (mm) | Preoperation | Follow-up | | *p* value |
| --- | --- | --- | --- | --- | --- |
|  |  |  | 90±30 Days 180±30 Days | |  |
| Group A | Aortic root | 25.83±2.14 | 23.67±2.09* | / | 0.042 |
|  | Proximal branch | 13.73±1.07 | 15.18±0.89 | / | 0.148 |
|  | Distal branch | 10.07±2.02 | 11.32±0.39 | / | 0.264 |
|  | Distal aortic arch | 19.93±2.74 | 22.00±2.25* | / | 0.031 |
|  | Distance between branches | 5.88±2.08 | 6.79±0.99 | / | 0.342 |
| Group B | Aortic root | 28.32±3.97 | / | 26.43±2.90 | 0.155 |
|  | Proximal branch | 13.60±0.52 | / | 14.88±0.50* | 0.001 |
|  | Distal branch | 9.95±1.57 | / | 11.98±0.86 | 0.067 |
|  | Distal aortic arch | 23.07±5.82 | / | 24.17±3.06 | 0.521 |
|  | Distance between branches | 5.84±1.07 | / | 7.11±1.01* | 0.010 |

*Results were significantly different from those before operation (*p* < 0.05).

**References**

[1] Lio A, Nicolò F, Bovio E, Serrao A, Zeitani J, Scafuri A *et al.* Total arch versus hemiarch replacement for type a acute aortic dissection: A single-center experience. Tex Heart Inst J 2016;43:488-95.

[2] Carrel T, Sundt TM, 3rd, von Kodolitsch Y and Czerny M. Acute aortic dissection. Lancet 2023;401:773-88.

[3] Iino K, Takago S, Saito N, Ueda H, Yamamoto Y, Kato H *et al.* Total arch replacement and frozen elephant trunk for acute type a aortic dissection. J Thorac Cardiovasc Surg 2022;164:1400-9.e3.

[4] Verhoye JP, Anselmi A, Kaladji A, Flécher E, Lucas A, Heautot JF *et al.* Mid-term results of elective repair of extensive thoracic aortic pathology by the evita open plus hybrid endoprosthesis only. Eur J Cardiothorac Surg 2014;45:812-7.

[5] Oda T, Minatoya K, Sasaki H, Tanaka H, Seike Y, Itonaga T *et al.* Adventitial inversion technique for type a aortic dissection distal anastomosis. J Thorac Cardiovasc Surg 2016;151:1340-5.

[6] Milewski RK, Szeto WY, Pochettino A, Moser GW, Moeller P and Bavaria JE. Have hybrid procedures replaced open aortic arch reconstruction in high-risk patients? A comparative study of elective open arch debranching with endovascular stent graft placement and conventional elective open total and distal aortic arch reconstruction. J Thorac Cardiovasc Surg 2010;140:590-7.

[7] Tokuda Y, Oshima H, Narita Y, Abe T, Araki Y, Mutsuga M *et al.* Hybrid versus open repair of aortic arch aneurysms: Comparison of postoperative and mid-term outcomes with a propensity score-matching analysis. Eur J Cardiothorac Surg 2016;49:149-56.

[8] Cochennec F, Tresson P, Cross J, Desgranges P, Allaire E and Becquemin JP. Hybrid repair of aortic arch dissections. J Vasc Surg 2013;57:1560-7.

[9] Jakob H, Dohle D, Benedik J, Jánosi RA, Schlosser T, Wendt D *et al.* Long-term experience with the e-vita open hybrid graft in complex thoracic aortic disease†. Eur J Cardiothorac Surg 2017;51:329-38.

[10] Shrestha M, Kaufeld T, Beckmann E, Fleissner F, Umminger J, Abd Alhadi F *et al.* Total aortic arch replacement with a novel 4-branched frozen elephant trunk prosthesis: Single-center results of the first 100 patients. J Thorac Cardiovasc Surg 2016;152:148-59.e1.

[11] Bozso SJ, White A, Nagendran J, Moon MC and Chu MWA. Hybrid aortic arch and frozen elephant trunk reconstruction: Bridging the gap between conventional and total endovascular arch repair. Expert Rev Cardiovasc Ther 2018;16:209-17.

[12] Lorenz V, Muzzi L, Tommasino G, Tucci E and Neri E. (s)ine: (soft-graft)-induced new entry tear after elephant trunk procedure. Interdiscip Cardiovasc Thorac Surg 2023;36.

[13] Chen LW, Dai XF, Lu L, Zhang GC and Cao H. Extensive primary repair of the thoracic aorta in acute type a aortic dissection by means of ascending aorta replacement combined with open placement of triple-branched stent graft: Early results. Circulation 2010;122:1373-8.

[14] Chen LW, Wu XJ, Dai XF, Liao DS, Li C, Wang QM *et al.* A self-adaptive triple-branched stent graft for arch repair during open type a dissection surgery. J Thorac Cardiovasc Surg 2015;149:1278-83.e1.

[15] Weigang E, Nienaber CA, Rehders TC, Ince H, Vahl CF and Beyersdorf F. Management of patients with aortic dissection. Dtsch Arztebl Int 2008;105:639-45.

[16] Elbatarny M, Stevens LM, Dagenais F, Peterson MD, Vervoort D, El-Hamamsy I *et al.* Hemiarch versus extended arch repair for acute type a dissection: Results from a multicenter national registry. J Thorac Cardiovasc Surg 2023, doi 10.1016/j.jtcvs.2023.04.012.

[17] Poon SS, Theologou T, Harrington D, Kuduvalli M, Oo A and Field M. Hemiarch versus total aortic arch replacement in acute type a dissection: A systematic review and meta-analysis. Ann Cardiothorac Surg 2016;5:156-73.

[18] Ma WG, Zhu JM, Zheng J, Liu YM, Ziganshin BA, Elefteriades JA *et al.* Sun's procedure for complex aortic arch repair: Total arch replacement using a tetrafurcate graft with stented elephant trunk implantation. Ann Cardiothorac Surg 2013;2:642-8.

[19] Chen LW, Dai XF, Wu XJ, Liao DS, Hu YN, Zhang H *et al.* Ascending aorta and hemiarch replacement combined with modified triple-branched stent graft implantation for repair of acute debakey type i aortic dissection. Ann Thorac Surg 2017;103:595-601.

[20] Chen LW, Lu L, Dai XF, Wu XJ, Zhang GC, Yang GF *et al.* Total arch repair with open triple-branched stent graft placement for acute type a aortic dissection: Experience with 122 patients. J Thorac Cardiovasc Surg 2014;148:521-8.

[21] Shen K, Tang H, Jing R, Liu F and Zhou X. Application of triple-branched stent graft for stanford type a aortic dissection: Potential risks. Eur J Cardiothorac Surg 2012;41:e12-7.

[22] Williams MJ, Utzinger U, Barkmeier-Kraemer JM and Vande Geest JP. Differences in the microstructure and biomechanical properties of the recurrent laryngeal nerve as a function of age and location. J Biomech Eng 2014;136:0810081-9.

[23] Kondov S, Beyersdorf F, Braun N, Höhn R, Schlett C, Rylski B *et al.* Morphology of the left subclavian artery: Implications for single-branched endovascular aortic arch repair. Eur J Cardiothorac Surg 2022;61:348-54.

[24] Yu B, Liu Z, Xue C, Liu J, Yang J, Jin Z *et al.* Total arch repair with open placement of a novel double-branched stent graft for acute type a aortic dissection: A single-centre experience with 21 consecutive patients. Interact Cardiovasc Thorac Surg 2019;28:262-9.

[25] An Z, Sun YY, Fan RX, Yu SQ, Zhu JM, Han QQ *et al.* Extensive arch repair with a novel two-branched stent graft in acute type a aortic dissection. Ann Thorac Cardiovasc Surg 2022;28:255-61.

[26] Sun X, Lu S, Yang S, Lai H, Chen H, Hong T *et al.* Open triple-branched stent graft placement for the surgical treatment of acute aortic arch dissection. J Cardiothorac Surg 2012;7:130.

[27] Qiao F, Su C, Han Q, Tan M, Wang J, Liu Y *et al.* Hybrid reconstruction of the aortic arch using a double-branched stent-graft in a canine model. J Invest Surg 2019;32:491-500.
