# Supplementary material for: Double-branched stent graft and four-stage deployment in total arch repair: safety and feasibility evaluation in porcine models
Source: Interdiscip Cardiovasc Thorac Surg. 2024 Mar 16;38(4):ivae049. doi: 10.1093/icvts/ivae049 (PMC11014789; doi:10.1093/icvts/ivae049)
Supplement: ivae049_Supplementary_Data [file ivae049_supplementary_data.zip › Supplementary Data.docx]

Supplementary Table 1. The exact value in the MATERIALS and METHODS section.

| Subject | Numerical Value |
| --- | --- |
| Animal Preparation |  |
| Sex | 4 males; 8 females |
| Age | 7 months (before stent implantation) |
| Body weight | 70-120 kg |
| Room temperature | 16-28 °C |
| Relative humidity | 40-70% |
| Fast | 48-hour |
| Water take inhibition | 8-hour |
| Sutaride | 5mg/kg |
| Atropine sulfate | 0.08 mg/kg |
| Propofol (induction) | 1-1.5 mg/kg |
| Propofol and Isoflurane (maintain) | 10 mg/kg/h |
| Operation Procedure |  |
| Retrograde cerebral perfusion | 5-8 ml/kg/min |
| Temperature rewarmed | 35 °C |
| U penicillin | 1.6 million |
| Cefoperazone/Sulbactam sodium | 1 g/d |
| Aspirin | 100 mg/d |
| Follow-Up |  |
| Follow-up endpoint | 90±30 d (Group A); 180±30 d (Group B) |
| Heparin sodium | 1.5 mg/kg |
| Potassium chloride | 60 mL, 15% |

Supplementary Table 2. Basic Information of Animals and Device Selection.

| Case Number | Number | Product Number | Group | Gender | Survival Days |
| --- | --- | --- | --- | --- | --- |
| 1 | PH-1 | IS3214112010010001 | B | Female | 178 |
| 2 | PH-2 | IS2413102010010001 | B | Male | 179 |
| 3 | PH-3 | IS2614122101010001 | B | Male | 189 |
| 4 | PH-4 | IS2412102101010001 | B | Female | 188 |
| 5 | PH-5 | IS2614102101010001 | B | Female | 184 |
| 6 | PH-6 | IS2214102101010001 | B | Female | 176 |
| 7 | PH-7 | IS2413102101010001 | A | Female | 84 |
| 8 | PH-8 | IS2214102101010002 | A | Female | 82 |
| 9 | PH-9 | IS2414102101010001 | A | Female | 97 |
| 10 | PH-10 | IS2214102101010003 | A | Male | 120 |
| 11 | PH-11 | IS2014102101010001 | A | Female | 120 |
| 12 | PH-12 | IS2614122101010002 | A | Male | 102 |

Supplementary Table 3. Results of Perioperative Characteristics in Group A and Group B

| Group | Project | Preoperation | Follow-up | |
| --- | --- | --- | --- | --- |
|  |  |  | 90±30 Days 180±30 Days | |
| Group A | Weight (Kg) | 91.75±13.37 | 114.77±5.19* | / |
|  | Temperature (°C) | 38.43±0.23 | 38.38±0.25 | / |
|  | Respiration (times/minute) | 16.17±1.07 | 15.67±1.59 | / |
|  | Heart Rate (times/minute) | 108.67±7.74 | 107±1.29 | / |
|  | Blood Pressure (mmHg) | 104.67±2.49 | 102.33±6.10 | / |
| Group B | Weight (Kg) | 86.95±9.06 | / | 119.50±1.50* |
|  | Temperature (°C) | 38.44±0.21 | / | 38.38±0.24 |
|  | Respiration (times/minute) | 15.29±1.03 | / | 15.26±1.48 |
|  | Heart Rate (times/minute) | 102.14±4.52 | / | 106.43±2.38 |
|  | Blood Pressure (mmHg) | 98.14±5.67 | / | 101.57±4.49 |

*Results were significantly different from those before the operation (*p* < 0.05).

Supplementary Table 4. Information on Laboratory Examinations for Group A and Group B.

| Group | Total Hemoglobin (g/dl) | |
| --- | --- | --- |
|  | Preoperation | After CPB |
| Group A | 9.68±1.57 | 7.78±1.20* |
| Group B | 9.40±1.29 | 8.32±0.25 |

*Results were significantly different from those before the operation (p < 0.05). CPB, cardiopulmonary bypass.
